# Potential value and research frontiers of virus in neuroinflammation: a bibliometric and visualized analysis

**DOI:** 10.3389/fimmu.2024.1390149

**Published:** 2024-07-03

**Authors:** Danyang Li, Minghua Wu

**Affiliations:** ^1^ Hunan Cancer Hospital/the Affiliated Cancer Hospital of Xiangya School of Medicine, Central South University, Changsha, Hunan, China; ^2^ The Key Laboratory of Carcinogenesis of the Chinese Ministry of Health, The Key Laboratory of Carcinogenesis and Cancer Invasion of the Chinese Ministry of Education, Cancer Research Institute, School of Basic Medicine, Central South University, Changsha, Hunan, China

**Keywords:** bibliometric analysis, neuroinflammation, virus, psychiatric disorders, VOSviewer, CiteSpace, hotspots

## Abstract

**Background:**

Neuroinflammation represents the immune response of the central nervous system to nerve injury, infection, toxin stimulation, or autoimmunity and is implicated in a wide range of neurological disorders. Viruses play a pivotal role as extrinsic biological drivers in neuroinflammation; however, numerous aspects remain unexplored. In this study, we employed bibliometric analysis to assess the current status of viral research in neuroinflammation and anticipate future research directions and emerging trends.

**Methods:**

Conduct a comprehensive search for scholarly publications within the Web of Science Core Collection database, with search terms on neuroinflammation and virus. Apply Microsoft Excel Office, Hiplot, R (version 4.3.1), VOSviewer (version 1.6.20) and CiteSpace (6.2.R6, advanced) software for the bibliometric analysis and visualization.

**Results:**

A total of 4230 articles and reviews on virus and neuroinflammation were identified, demonstrating a consistent upward trend over time. The United States was the country that contributed the most publications. Approximately 22274 authors from 4474 institutions contributed to the research. Johns Hopkins University leads with the highest number of publications and citations. The top three authors with the most published articles on this field are Power, C., Lane, T. E., and Buch, S. The Journal of Neuroinflammation is the most authoritative choice for researchers. The main research focuses in this field include multiple sclerosis, Parkinson’s disease, blood-brain barrier, COVID-19, Alzheimer’s disease, gene therapy. In recent years, stress have emerged as hot keywords, particularly depression, human immunodeficiency virus-associated neurocognitive disorders, blood-brain barrier, gut microbiota related directions, indicating a potential shift in research focus.

**Conclusion:**

Research on the virus and neuroinflammation has attracted increasing attention in the past decade. European and American countries have been pivotal in conducting research on virus and neuroinflammation, while China has produced a significant number of publications, its impact is still limited. Stress is likely to emerge as the next area of focus in this field. The association and regulation between viral infection and psychiatric disorders are not fully understood, and further research is needed to explore the role of neuroinflammation caused by different types of viral infection and psychiatric disorders.

## 1 Introduction

Neuroinflammation is an immune response activated by glial cells in the central nervous system (CNS), typically occurring in response to nerve injury, infection, toxin stimulation, or autoimmunity (1). This response is implicated in nearly all neurological disorders. Under normal physiological conditions, neuroinflammation can effectively clear or inhibit harmful substances, aid the body’s defense against pathogen invasion, and maintain internal homeostasis (2). However, excessive activation of neuroinflammation upon stimulation can lead to neuronal damage and exacerbate disease progression (3). Persistent inflammatory responses can activate glial cells (primarily microglia and astrocytes) within the CNS that serve functions such as immune surveillance and danger signal recognition while playing a pivotal role in maintaining CNS homeostasis and mediating neuroinflammatory responses (1, 4). Furthermore, recent studies have highlighted the significant contribution of peripheral systemic inflammation (5). For instance, disruption of tight junctions or endothelial cell damage impairs blood-brain barrier (BBB) function allowing infiltration of peripheral proinflammatory factors, immune cells, and harmful substances into the brain parenchyma (6, 7). Additionally, peripheral nerves transmit inflammatory signals from periphery to brain through receptors for inflammatory factors. These inflammatory factors and signals can directly or indirectly activate microglia and astrocytes leading to an inflammatory response within the CNS (8). Currently, the significance of neuroinflammation in the field of neurology is increasingly evident. Apart from well-established neuroinflammatory disorders like multiple sclerosis (MS), neuroinflammation also assumes a pivotal role in numerous ostensibly non-inflammatory neurological conditions, including Alzheimer’s disease (AD), Parkinson’s disease (PD), schizophrenia, stroke, and Glioblastoma (9–13).

Due to the concept of “neuroinflammation”, the brain is no longer considered a closed area. There is a bidirectional and complex interaction between the nervous system and the immune system, which plays a pivotal role in perceiving internal and external environmental stimuli as well as maintaining physiological homeostasis. Due to the prolonged parasitic nature of viruses within the human body, their wide-ranging infectivity, and limited targeted treatment options in certain cases, research on virus infection and neuroinflammation has progressively emerged as a field of interest among neuropathology researchers. The earliest studies in this field focused on viral meningitis and viral encephalitis caused by enterovirus, mumps virus, herpes simplex virus, and adenovirus (14, 15), and the incidence of both is increasing year by year, with high mortality and disability rates. Due to the varying regions of brain inflammation invasion, distinct clinical manifestations arise which can lead to different degrees of neurological sequelae. This poses a significant threat to human health while also resulting in substantial economic losses. Recent investigations have gradually unveiled that viral infections substantially elevate the risk of neurodegenerative diseases such as AD and PD. Multi-omics studies have identified elevated genomic DNA levels of human herpesviruses 6A and 7 within the brains of AD patients compared with cognitively normal controls; moreover, viral abundance correlates with transcriptomic features associated with amyloid-β (Aβ) processing (16, 17). Furthermore, amidst the outbreak of Corona Virus Disease 2019 (COVID-19), research has demonstrated persistent inflammatory responses within the brain tissue of individuals with mild COVID-19 infection; additionally, COVID-19 infection significantly heightens the risk for psychiatric disorders (18). Although there is ample evidence supporting the crucial role of viral infections in neuroinflammatory disorders’ development and progression, research on non-inflammatory neurological disorders mainly focuses on clinically relevant studies. Therefore, conducting an extensive investigation into neuroinflammation induced by viral infections will enhance our understanding of their involvement in non-inflammatory neurological disorders and refine our comprehension of pathological processes underlying CNS diseases, ultimately suggesting personalized treatment strategies for these diseases.

Bibliometrics emerged in the early 20th century and became an independent discipline in 1969, which has been widely used in literature analysis (19). It is a quantitative method used to describe and analyze the dynamics and progress of a certain discipline or research field. In the analysis process, detailed information such as countries, institutions, journals, authors, keywords, and references can be obtained (20). With the assistance of computer technology, the results of literature analysis can be visually represented to explore information relationships effectively.

To date, despite the extensive research conducted on the relationship between viruses and neuroinflammation, there remains a lack of clarity regarding the overall research trends in this area. In order to address this gap, this study aims to comprehensively explore the research trends and emerging hotspots concerning viruses and neuroinflammation from a bibliometric perspective, thereby providing guidance for future investigations into non-inflammatory neurological disorders caused by viral infections.

## 2 Materials and methods

### 2.1 Data source and search strategy

**Figure 1:**
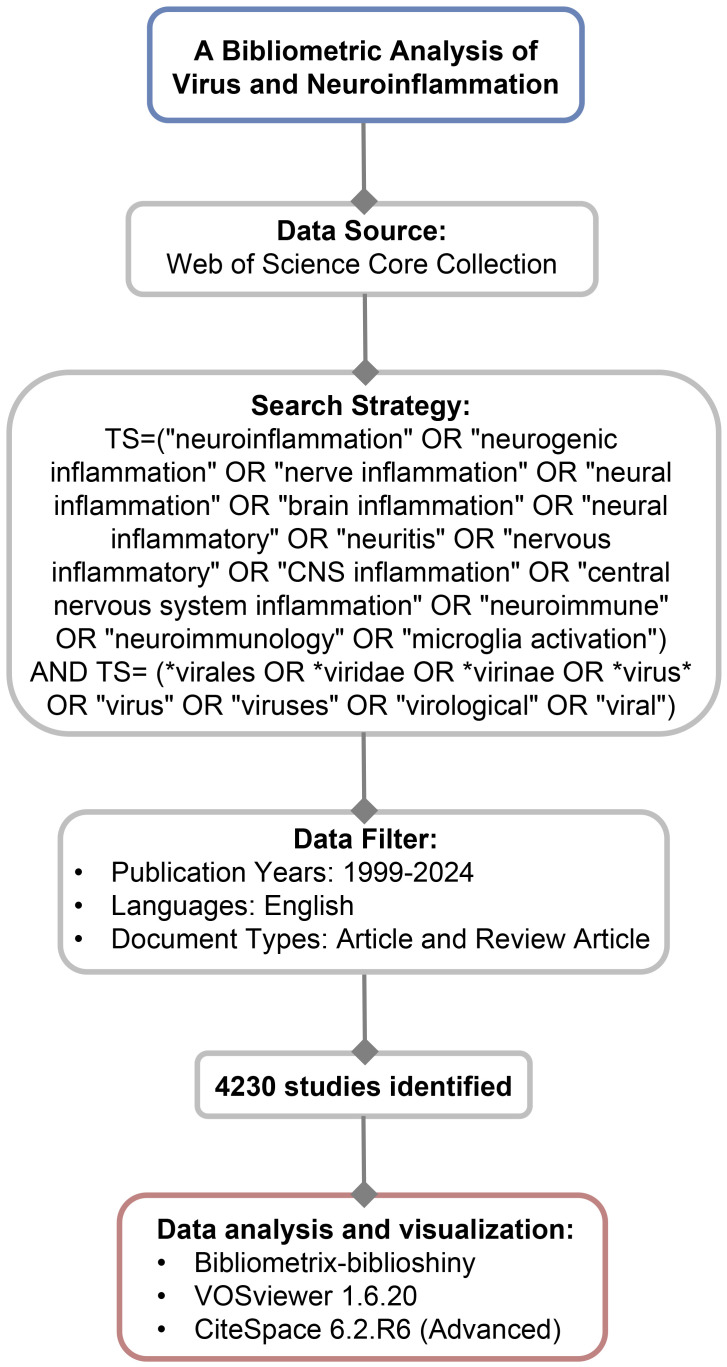
Flowchart of data collection and study design.

As a high-quality digital literature resource database covering various fields, Web of Science (WOS) has been accepted by many researchers and is the most suitable database for bibliometric analysis (21, 22). We conducted a comprehensive search for scholarly publications within the WOS Core Collection (WOSCC) database. The search strategy was set as the following: TS = (“neuroinflammation” OR “neurogenic inflammation” OR “nerve inflammation” OR “neural inflammation” OR “brain inflammation” OR “neural inflammatory” OR “neuritis” OR “nervous inflammatory” OR “CNS inflammation” OR “central nervous system inflammation” OR “neuroimmune” OR “neuroimmunology” OR “microglia activation”) AND TS = (*virales OR *viridae OR *virinae OR *virus* OR “virus” OR “viruses” OR “virological” OR “viral”). The retrieved data were collected on January 31, 2024, to avoid any potential deviation due to daily updates. The publications included in this study are categorized as either Article or Review Article and are written in the English language. A total of 4230 records were exported as the format of “plain text file”, and then recorded as “full record and cited references”. The flowchart of the study is shown in **Figure 1**.

### 2.2 Data analysis and visualization

This study used Microsoft Excel Office, Hiplot, R (version 4.3.1), VOSviewer (version 1.6.20), and CiteSpace (6.2.R6, advanced) as the software tools for organizing, analyzing and visualizing data.

Microsoft Excel Office was used in this study to organize relevant data including the number of publications and citation. Hiplot (https://hiplot.com.cn) was used in this study to map the global geographic distribution.

Bibliometrix package in R software provides a set of tools for quantitative research in bibliometrics and scientometrics (23). And biblioshiny is a shiny app that presents the web interface of bibliometrix, which supports scholars to easily use the main features of bibliometrix (24). The tool can perform data importing, conversion to data frame collection and data filtering. Additionally, it can analyze and visualize the data based on three different level metrics (sources, authors and documents) and three structures of Knowledge (conceptual structure, intellectual structure and social structure). In this study, it was used to summarize the overview of the data and convert it into a dataset that can be used for R analysis and analyze the annual number of documents and citations.

VOSviewer is a free Java-based software developed in 2009 by van Eck and Waltman of Leiden University in the Netherlands (25). VOSviewer uses a data standardization method based on probability theory to analyze each information of the literature and visualize the results, which has been applied to research in many fields. VOSviewer provides three kinds of visualization views: network visualization, overlay visualization, and density visualization, which have the advantages of beautiful images and easy interpretation. The subject terms and keywords obtained from the database can be used to describe the research status and internal correlation in this field through cooperative network analysis, co-word analysis and cluster analysis. Therefore, VOSviewer was used to conduct a statistical analysis of relevant research countries, institutions, journals, authors, keywords, and references, summarize hot research, and look forward to the research frontier.

CiteSpace is developed by Professor Chaomei Chen of Drexel University in the United States (26). It is mainly used for literature review and summary, and for visual quantitative analysis of current data. It is a widely used bibliometric analysis tool for citation space. In addition to having the same functions as VOSviewer, CiteSpace can also extract information from titles, keywords and abstracts and generate cluster labels according to log-likelihood rate (LLR), latent semantic indexing (LSI) and mutual information (MI) algorithms (27). In addition, it can also find the research hotspots and heat in different periods through the emergence of keywords, so as to analyze the development trend of the field. VOSviewer was used in this study to perform the dual-map overlay of journals, the cluster analysis, timeline visualization and burst analysis of references and keywords.

This study followed the guideline for reporting bibliometric reviews of the biomedical literature (BIBLIO) (28).

## 3 Results

### 3.1 Global trend in publication outputs and citations

**Figure 2:**
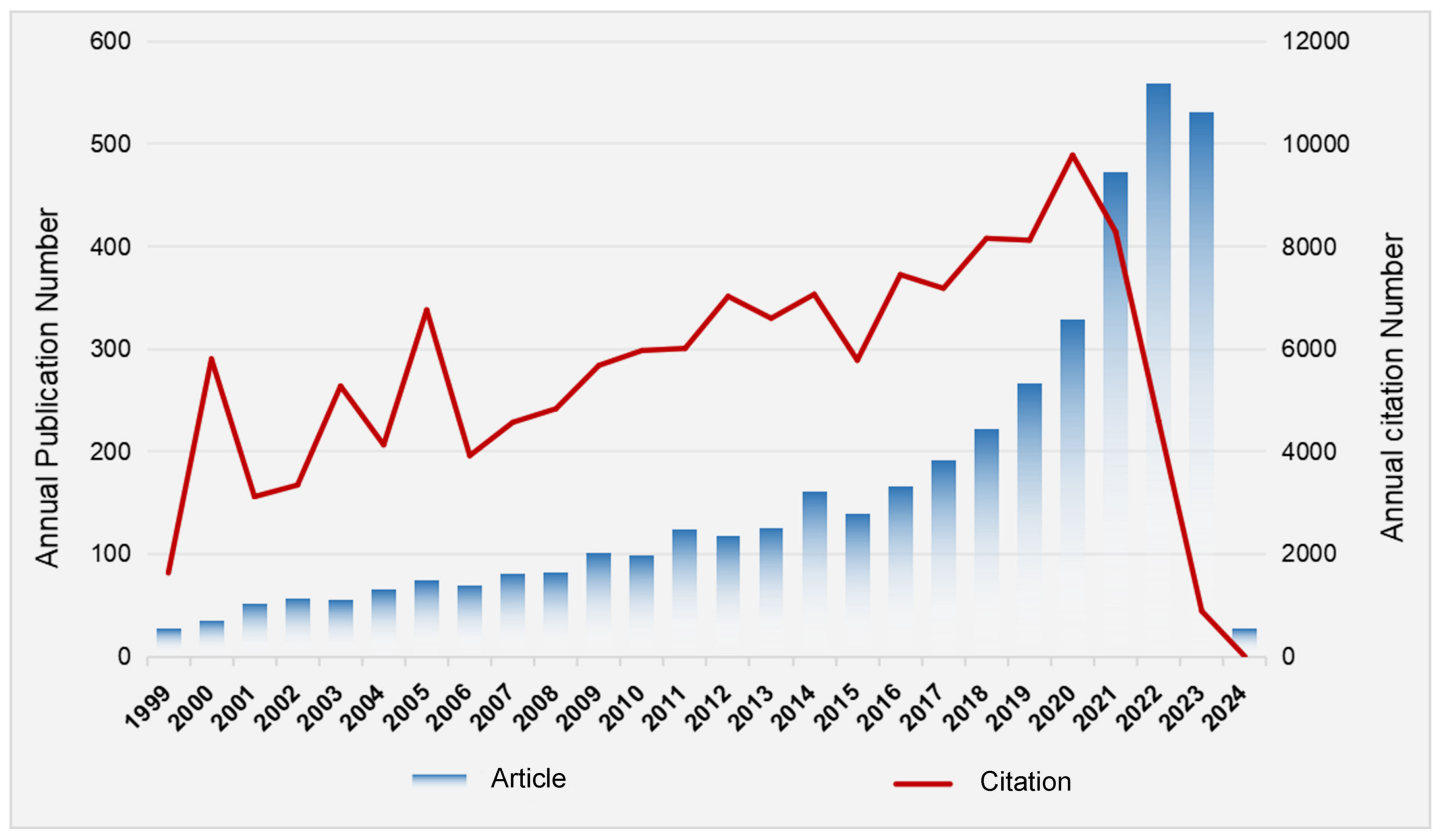
Trends in the growth of publications and the number of citations. The number of publications and citation frequency for each year from 1999 to the January 2024 showed the steady growth trend.

The 4230 publications utilized in this study were written by 22274 authors from 4474 institutions in 106 countries, published in 1046 journals, and cited 191063 references from 13107 journals. **Figure 2** illustrates the annual publication volume and annual citation frequency of related articles from 1999 to the January 2024. Overall, there has been a consistent upward trend in the number of annual publications pertaining to viruses and neuroinflammation, with a notable surge observed since the year 2021. The year with the highest number of publications is 2022, with 559 articles. With the increase of the number of publications, the annual citation frequency of relevant literature showed a rugged upward trend. The number of citations in 2022 was as high as 9780. This indicates that the research concerning virus and neuroinflammation has garnered substantial attention and emerged as a prominent area for investigation within recent years.

### 3.2 Distribution of countries/regions

**Figure 3:**
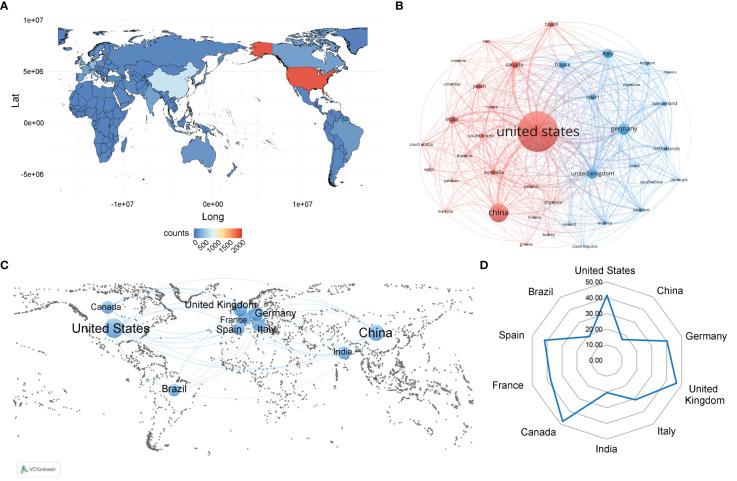
Analysis of country/region. **(A)** Geographical distribution of global output. The volume of publications is represented by color variation. **(B)** Visual cluster analysis of cooperation among countries. The nodes of different colors represent the countries/regions with different clusters, and the thickness of the lines indicates how closely countries cooperate. **(C)** Geographical distribution and the co-authorship network of the top 10 productive countries. **(D)** Radar chart of the average cited frequency of the top 10 productive countries.

There were 106 countries involved in publications on the virus and neuroinflammation. The geographical distribution of these countries is shown in **Figure 3A**, and the volume of publications is represented by color variation, which shows that the countries involved in this field are mainly distributed in North America, East Asia, and Europe. **Figure 3B** shows the international cooperation relationships in the research on virus and neuroinflammation. The connections between nodes reflects the collaborative relationship between individual countries and regions, and the thickness of the links is positively correlated with the depth of collaboration (29). It is worth noting that the links between countries/regions are mainly concentrated between the United States and other countries, whereas research collaborations among other countries were scattered. To further analyze the highly productive countries in this field, **Table 1** shows the top 10 countries/regions in terms of number of publications, and **Figure 3C** visualizes the geographical distribution and collaboration. The United States emerged as the leading contributor with 1,908 articles published, followed by China with 649 articles and Germany with 325 articles. In terms of citations received, the United States garnered the highest count with 79247 citations, followed by Germany with 13055 citations and the United Kingdom with 12287 citations. Although United States has the highest number of publications, the average number of citations per article (N = 41.53) is lower than that of Canada (N = 47.86), the United Kingdom (N = 46.54) and Spain (N = 41.74). At the same time, although China has a high number of publications and citations, its average number of citations per article (N = 16.55) is the lowest among the 10 countries, indicating that the academic influence of Chinese scholars is low, and it is still necessary to publish higher quality, more innovative and widely recognized academic papers in the professional field (**Figure 3D**).

**Table 1:** The top 10 most productive countries.

Rank	Country	Counts	Citations
1	United States	1908	79247
2	China	649	10739
3	Germany	325	13055
4	United Kingdom	264	12287
5	Italy	236	7278
6	India	189	3851
7	Canada	182	8711
8	France	177	6682
9	Spain	135	5635
10	Brazil	132	2490

### 3.3 Analysis of institutions and authors

**Figure 4:**
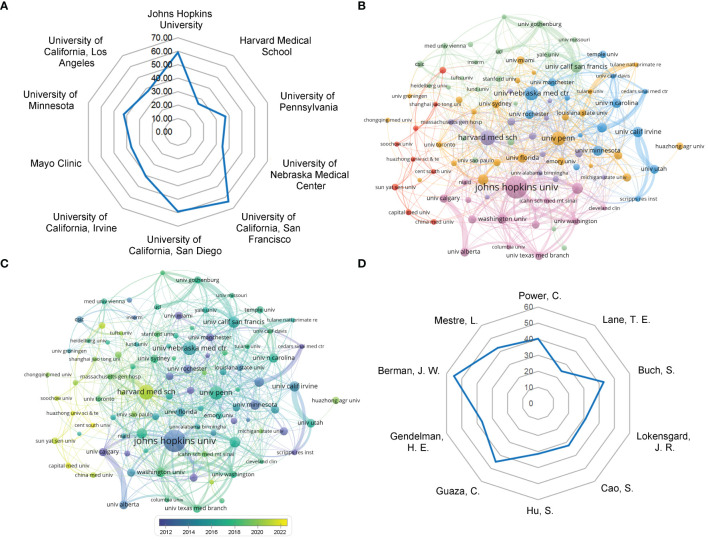
Analysis of institutions and authors. **(A)** Radar chart of the average cited frequency of the top 10 institutions. **(B)** Visual cluster analysis of cooperation among institutions. The nodes of different colors represent the institutions with different clusters, and the thickness of the lines indicates how closely institutions cooperate. **(C)** Timeline visualization of cooperation among institutions. The different colors represent the time at which the institution began the relevant study. **(D)** Radar chart of the average cited frequency of the top 10 authors.

Approximately 22274 authors from 4474 institutions contributed to the research on virus and neuroinflammation. **Table 2** and **Figure 4A** show the citations and the average number of citations per article of the top 10 institutions in terms of publication volume. Johns Hopkins University leads with the highest number of publications and citations, whereas University of California, San Francisco has the highest average number of citations. Also, the top 10 institutions were in the United States, which demonstrated the high interest of the United States institutions in the study of virus and neuroinflammation and highlighted their important position and contribution to this field of research. To further investigate collaboration between institutions, we performed a co-authorship analysis of all publications. It is showed that the division of institutions into 6 clusters based on an occurrence frequency and cooperative relationships (**Figure 4B**). The institutions with close collaboration were mostly in the United States, further signifying the robustness of related research in the United States. Among them, Johns Hopkins University has the strongest collaboration with other institutions. According to **Figure 4C**, the United States institutions such as Johns Hopkins University have published relevant articles since around 2012. The institutions from China (the red cluster in **Figure 4B**) have been active in the virus and neuroinflammation research in recent years (yellow color).

**Table 2:** The top 10 most productive institutions.

Rank	Institutions	Counts	Citations
1	Johns Hopkins University	102	6027
2	Harvard Medical School	66	1708
3	University of Pennsylvania	60	2228
4	University of Nebraska Medical Center	59	2053
5	University of California, San Francisco	48	3066
6	University of California, San Diego	47	2777
7	University of California, Irvine	44	1791
8	Mayo Clinic	43	1567
9	University of Minnesota	42	1779
10	University of California, Los Angeles	41	1545

The top three authors with the most published articles on the virus and neuroinflammation are Power, C. from the University of Alberta, Lane, T. E. from University of Utah Salt Lake City, and Buch, S. from the University of Nebraska Medical Center (**Table 3**). Berman, J. W. from the Albert Einstein College of Medicine has the highest average number of citations (**Figure 4D**). Her research focuses on the mechanisms of human immunodeficiency virus (HIV) infection and BBB penetration (30, 31). **Supplementary Figure 1** shows the maps of cooperation between authors; the minimum number of papers per author was set as 7. Of the remaining 130 authors, there were few links, indicating inadequate collaboration between research teams/laboratories conducting relevant research in the field.

**Table 3:** The top 10 most productive authors.

Rank	Author	Counts	Affiliations	Citations	Average Citation/Publication
1	Power, C.	28	University of Alberta	1128	40.29
2	Lane, T. E.	27	University of Utah Salt Lake City	670	24.81
3	Buch, S.	21	University of Nebraska Medical Center	903	43
4	Lokensgard, J. R.	20	University of Minnesota	622	31.1
5	Cao, S.	20	Huazhong Agricultural University	648	32.4
6	Hu, S.	19	University of Minnesota	597	31.42
7	Guaza, C.	19	Consejo Superior de Investigaciones Científicas	854	44.95
8	Gendelman, H. E.	19	University of Nebraska Medical Center	698	36.74
9	Berman, J. W.	18	Albert Einstein College of Medicine	993	55.17
10	Mestre, L.	17	Consejo Superior de Investigaciones Científicas	727	42.76

### 3.4 Distribution of journals

**Figure 5:**
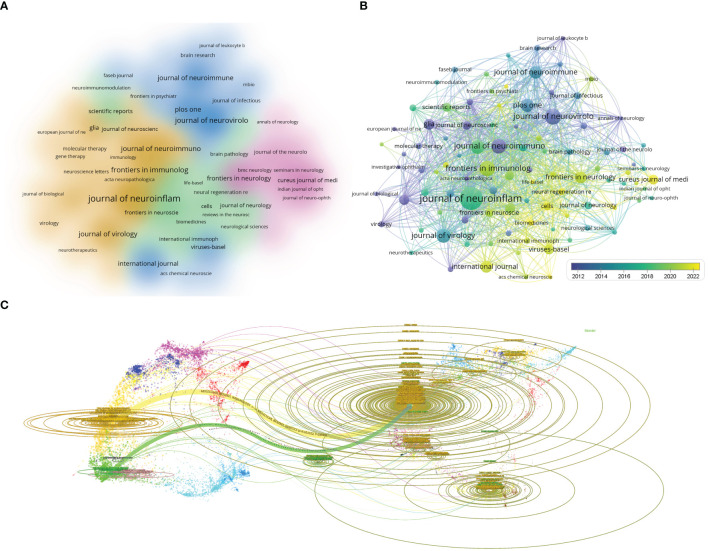
Analysis of journals. **(A)** Visual cluster analysis among journals. The density map of different colors represent the journals with different clusters. **(B)** Timeline visualization among Journals. The different colors represent the time when the journal began to publish relevant studies. **(C)** The dual-map overlap of journals. Citing journals are on the left, cited journals are on the right, and colored paths indicate citation relationships.

The articles on virus and neuroinflammation research were published across 1046 journals. **Table 4** displays the top 10 journals with the greatest number of publications and their recent impact factor (IF). The Journal of Neuroinflammation leads with 201 articles, followed by Journal of Neurovirology with 103 articles, and Frontiers in Immunology with 96 articles. According to the density map of the journals, the journals that published articles on this field were mainly divided into four categories: neuroimmunology, neurology, virology and comprehensive (**Figure 5A**). And in recent years, articles in this field tend to be published in comprehensive journals (**Figure 5B**). The dual-map overlay of journals indicates the position of the research topic in the mainstream research subject classification, showing the cited trajectory as well as the change of research center. Each dot on the graph represents a journal, with the citing chart on the left and the cited chart on the right. The length of the ellipse indicates the number of authors, the width of the ellipse indicates the number of publications, and the trajectory indicates the relationship between interdisciplinary classifications. Citing journals are mainly from MOLECULAR, BIOLOGY, IMMUNOLOGY, MEDICINE, MEDICAL, CLINICAL, and NEUROLOGY. The cited journals are mainly from MOLECULAR, BIOLOGY, GENETICS, PSYCHOLOGY, EDUCATION, and SOCIAL (**Figure 5C**).

**Table 4:** The top 10 most productive journals.

Rank	Journal	Counts	Citations	IF
1	Journal of Neuroinflammation	201	6090	9.3
2	Journal of Neurovirology	103	2514	3.2
3	Frontiers in Immunology	96	1936	7.3
4	Journal of Neuroimmunology	89	2270	3.3
5	Journal of Virology	83	2671	5.4
6	Brain Behavior and Immunity	77	2705	15.1
7	Plos One	73	3265	3.7
8	Journal of Neuroimmune Pharmacology	67	2367	6.2
9	International Journal of Molecular Sciences	58	807	5.6
10	Frontiers in Neurology	58	690	3.4

### 3.5 Analysis of research hotspots

#### 3.5.1 Most cited articles and cited authors

**Table 5** shows the top 10 most cited articles on the virus and neuroinflammation. All of them were published in journals classified as Q1 and with great influence. The most frequently cited article is ‘‘ Neurologic Manifestations of Hospitalized Patients with Coronavirus Disease 2019 in Wuhan, China ‘‘ which focuses on the neurologic manifestations of patients with COVID-19 (32). In general, research in this field mainly focused on HIV-associated neurocognitive disorders (HAND) before 2020, and COVID-19 and neuroinflammation after 2020. In addition, there are 283 cited authors, and we listed the top 10 based on different countries (**Table 6**). The number of cited authors was ranked by country as follows: the United States, China, Germany, Canada, South Korea, India, Spain, etc.

**Table 5:** The top 10 most cited references.

Rank	First author	Journal	DOI	Year	IF(2023)
1	Mao, L.	JAMA Neurology	DOI 10.1001/jamaneurol.2020.1127	2020	29.0	
2	Heaton, R. K.	Neurology	DOI 10.1212/wnl.0b013e318200d727	2010	9.9	
3	Antinori, A.	Neurology	DOI 10.1212/01.wnl.0000287431.88658.8b	2007	9.9	
4	Matschke, J.	Lancet Neurology	DOI 10.1016/s1474-4422(20)30308-2	2020	48.0	
5	González-Scarano, F.	Nature Reviews Immunology	DOI 10.1038/nri1527	2005	100.3	
6	Meinhardt, J.	Nature Neuroscience	DOI 10.1038/s41593-020-00758-5	2021	25.0	
7	Kaul, M.	Nature	DOI 10.1038/35073667	2001	64.8	
8	Hoffmann, M.	Cell	DOI 10.1016/j.cell.2020.02.052	2020	64.5	
9	Helms, J.	New England Journal of Medicine	DOI 10.1056/nejmc2008597	2020	158.5	
10	Moriguchi, T.	International Journal of Infectious Diseases	DOI 10.1016/j.ijid.2020.03.062	2020	8.4	

**Table 6:** The top 10 most cited authors from each country.

Country	Author	Counts	Citations	Average Citation/Publication
United States	Sacktor, N.	5	1053	211
	Harms, A.S.	8	1045	131
	Standaert, D.G.	8	1004	126
	Volsky, D.J.	7	858	123
	McArthur, J.C.	11	1214	110
	Valcour, V.	5	505	101
	Brundin, P.	5	470	94
	Smeyne, R.J.	5	457	91
	Gendelman, H.E.	7	635	91
	Nath, A.	6	541	90
Germany	Arbusow, V.	7	634	91
	Strupp, M.	8	654	82
	Gerhauser, I.	5	312	62
	Tumani, H.	7	401	57
	Brandt, T.	10	461	46
	Bauer, J,	8	361	45
	Chhatbar, C.	6	267	45
	Strupp, M.	14	597	43
	Kaeufer, C.	5	213	43
	Kalinke, U.	7	281	40
Canada	Power, C.	8	619	77
	Ellestad, K.K.	5	367	73
	Maingat, F.	7	349	50
	Silva, C.	6	297	50
	Branton, W.G.	7	309	44
	Power, C.H.	28	1128	40
	Noorbakhsh, F.	5	191	38
	Hollenberg, M.D.	5	183	37
	Cohen, E.A.	7	243	35
	Zhu, Y.	5	149	30
China	Huang, Y.L.	6	450	75
	Yao, H.H.	8	456	57
	Zhu, B,B,	6	339	57
	Chen, S.Y.	5	277	55
	Raung, S.L.	6	289	48
	Song, Y.F.	6	273	46
	Ou, Y.C.	7	318	45
	Cui, M.	10	448	45
	Chen, C.J.	8	336	42
	Liao, S.L.	8	336	42
India	Periyasamy, P.	8	399	50
	Basu, A.	17	765	45
	Vrati, S.	6	210	35
	Banerjee, A.	6	185	31
	Mishra, R.	6	149	25
	Banerjea, A.C.	5	105	21
	Das sarma, J.	17	331	19
	Jha, N.K.	5	52	10
Spain	Hernangomez, M.	5	289	58
	Docagne, F.	8	435	54
	Mecha, M.	13	660	51
	Guaza, C.	19	854	45
	Mestre, L.	17	727	43
	Feliu, A.	10	416	42
	Carrillo-Salinas, F.J.	5	199	40
South Korea	Suk, K.	5	124	25
	Kim, S.B.	7	166	24
	Kim, J.H.	7	157	22
	Uyangaa, E.	7	149	21
	Choi, J.Y.	9	177	20
	Eo, S.K.	9	177	20
	Kim, K.	8	145	18
	Park, S.Y.	6	97	16
	Hossain, F.M.A.	5	78	16
	Patil, A.M.	7	102	15

#### 3.5.2 Co-cited references and references bursts

**Figure 6:**
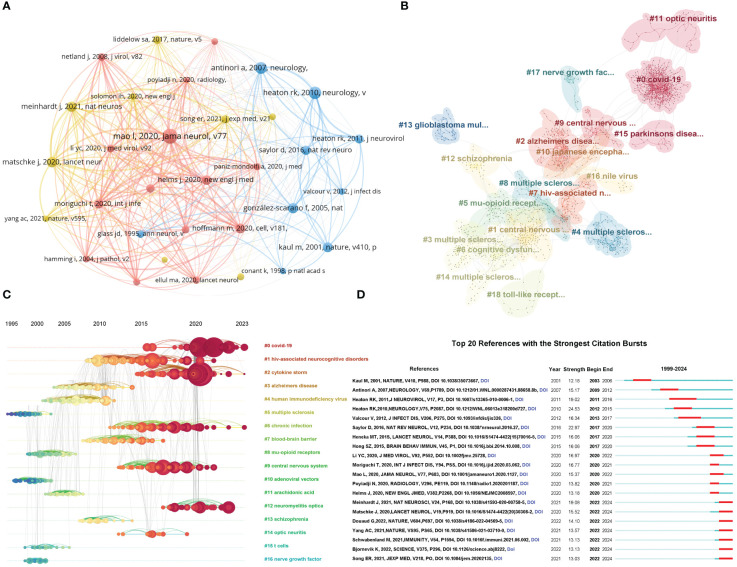
Analysis and network visualization of reference co-citation. **(A)** Visual analysis of co-cited references. The nodes of different colors represent the co-cited references with different clusters. **(B)** Cluster analysis of co-cited references. The nominal terms of these clusters are extracted from the keywords of the cited references by the LSI algorithm and clustered into 19 clusters. **(C)** Timeline distribution of the clusters. Each horizontal line represents a cluster. The smaller the number is, the larger the cluster, with #0 being the largest cluster. Nodes size reflects co-citation frequency, and the links between nodes indicate co-citation relationships. Nodes occurrence year is the time when they were first co-cited. **(D)** The top 20 references with the strongest citation bursts.

Co-citation analysis is a method to measure the relevance between academic papers, which can not only reflect the influence of articles in the field, but also reflect the hot research directions in the field. We further constructed a network visualization of co-cited references (**Figure 6A**) and conducted cluster analysis, which led to the identification of 19 cluster modules (**Figure 6B**). The nominal terms of these clusters are extracted from the keywords of the cited articles by the latent semantic indexing (LSI) algorithm. By studying the references corresponding to each node category, the mainly intellectual base of current research on virus and neuroinflammation was summarized as follows: Cluster 0 (COVID-19): studies that investigated the neurological manifestations and neuro-immune interactions in patients with COVID-19; Cluster 1 (central nervous system): studies that investigated the immune resistance of the central nervous system under viral infection; Cluster 2 (Alzheimer’s disease): studies that investigated the mechanism by which viral infection produces inflammatory triggers leading to Alzheimer’s disease; Cluster 3 (multiple sclerosis): studies that investigated the mechanism of virus and neuroimmune in the occurrence and development of multiple sclerosis. Furthermore, we visualized the clustering timeline (**Figure 6C**) and identified multiple sclerosis, mu-opioid receptors, adenoviral vectors, arachidonic acid, T cells and nerve growth factor as the focus of early research on virus and neuroinflammation. With the outbreak of COVID-19, related topics in this field such as cytokine storm, chronic infection, central nervous system, and neuromyelitis optica have been pushed to a research climax. **Figure 6D** shows the top 20 references with the strongest citation bursts. The strongest citation burst was for the 2010 article ‘‘ HIV-associated neurocognitive disorders persist in the era of potent antiretroviral therapy: CHARTER Study ‘‘ (33). This article has an intensity of 24.53. In recent years, the cited literature mainly studies neurological symptoms of patients with COVID-19 and reveals neuroinflammation with distinct microanatomical microglia-T-cell interactions (34).

#### 3.5.3 Keywords analysis of research hotspots

**Figure 7:**
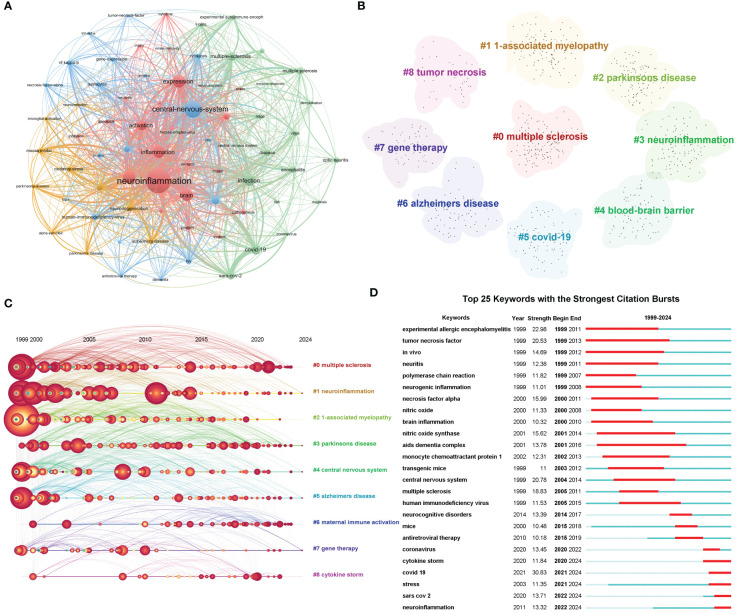
Analysis and network visualization of the research hotspots. **(A)** Network visualization map of keywords co-occurrence. The nodes of different colors represent the keywords with different clusters. **(B)** Cluster Analysis of keywords. The nominal terms of these clusters are extracted from the keywords of the publications by the LSI algorithm and clustered into 9 clusters. **(C)** Timeline distribution of cluster analysis of keywords. Nodes occurrence year is the time when they first appeared. **(D)** The top 25 keywords with the strongest citation bursts.

Keywords summarize the core and essence of a paper, and research hotspots in a scientific field can be found through keyword co-occurrence analysis. VOSviewer was used to draw the keyword co-occurrence network view, and 69 key keywords with a frequency greater than or equal to 80 were selected for visualization (**Figure 7A**). The frequency of keywords was positively correlated with the size of the circle node. Based on the co-occurrence network, the research strategy in this field is to explore the mechanism by which viral infection promotes neuroinflammation in the central nervous system by triggering inflammatory factors such as NF-κB/TNF-α, and then leading to diseases at the *in vitro* and *in vivo* levels. Then we performed cluster analysis through CiteSpace using the same strategy as the co-cited references and obtained 9 clusters, which were multiple sclerosis, 1-associated myelopathy, Parkinson’s disease, neuroinflammation, blood-brain barrier, covid-19, Alzheimer’s disease, gene therapy and tumor necrosis (**Figure 7B**). These findings highlight the key research topics in this field. Furthermore, we also conduct a timeline visualization analysis of keywords, which can show the dynamic evolution path of the research hotspots represented by the keywords, and explore the time characteristics and the rise and fall process of the research field reflected by the aggregation of hot keyword research (**Figure 7C**). From the evolution of keywords, large-scale studies in multiple sclerosis, neuroinflammation, 1-associated myelopathy, central nervous system, and Alzheimer’s disease were conducted in this field as early as 1999. In the following research showed an annual average, maternal immune activation and cytokine storm intensively entered the research field in 2020. In order to better understand the research hotspots of sudden outbreaks in the field of viruses and neuroinflammation, we conducted a burst analysis on keywords. **Figure 7D** shows the top 25 keywords with the strongest citation bursts. It can be seen that “covid-19” has the highest burst strength and may still have high intensity research in 2024. At the same time, we also noticed that “stress” from 2021 and “neuroinflammation” from 2022 have become the recent research upsurge, indicating that viruses, stress and neuroinflammation may become the new research hotspot in this field.

### 3.6 Virus, neuroinflammation and psychiatric disorders

**Figure 8:**
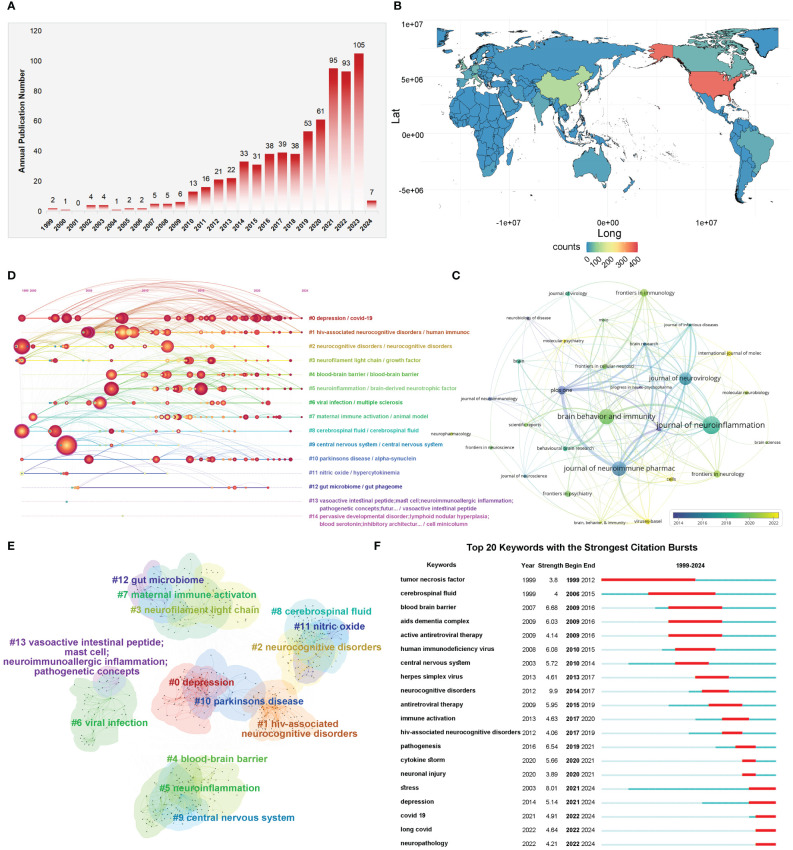
Analysis and network visualization of research on viruses, neuroinflammation, and psychiatric disorders. **(A)** Trends in the growth of publications. **(B)** Geographical distribution of global output. **(C)** Timeline visualization among Journals. The different colors represent the time when the journal began to publish relevant studies. **(D)** Timeline distribution of cluster analysis of keywords. Nodes occurrence year is the time when they first appeared. **(E)** Cluster Analysis of keywords. The nominal terms of these clusters are extracted from the keywords of the publications by the LSI algorithm and clustered into 13 clusters. **(F)** The top 20 keywords with the strongest citation bursts.

Based on the above findings and the global mental health reconstruction project in the context of COVID-19, we would like to take a deeper look at the current research on virus, neuroinflammation, and psychiatric disorders. According to the above search strategy, we added some conditions “TS= (mental* OR psychiatr* OR neuropsych* OR depressi* OR MDD OR anxi* OR “bipolar disorder” OR mania OR manic OR “mood disorder” OR “affective disorder” OR “feeding and eating disorder” OR anorexia OR “eating disorder” OR “neurocognitive disorders” OR “neurodevelopmental disorder” OR “personality disorder” OR schizophren* OR schizoaffect* OR psychotic OR psychosis OR “sleep wake disorders” OR “trauma and stressor related disorders” OR post-traumatic* OR PTSD OR autis* OR “attention deficit” OR ADHD OR “obsessive compulsive” OR OCD). After screening, 697 articles related to virus, neuroinflammation, and psychiatric disorders were included. In terms of global trends in publication output, the number of annual publications in this field has shown a steady growth trend, with a sharp increase since 2021 (**Figure 8A**). The United States leads with the highest number (n=366), followed by China (n=97) and the United Kingdom (n=52) (**Figure 8B**). According to overlay visualization of the Journal in which the article was published, the most articles in this field were published on The Journal of Neuroinflammation, and Molecular Psychiatry and Viruses-Basel has gained popularity for submissions in recent years (**Figure 8C**). Cluster analysis, timeline visualization, and burst analysis of keywords revealed that recent studies focused on depression, covid-19, HIV-associated neurocognitive disorders, neurofilament light chain, blood-brain barrier, hypercytokinemia, maternal immune activation, gut microbiota, stress, neuropathology and other directions, indicating that these areas are potential future research frontiers in the field of the virus, neuroinflammation and psychiatric disorders (**Figures 8D–F**).

## 4 Discussion

### 4.1 General information

This study conducted a comprehensive literature search of virus and neuroinflammation publications from January 1, 1999, to January 31, 2024, in the WOSCC database. After excluding publications that did not meet the predefined inclusion criteria, this bibliometric analysis encompassed a total of 4230 English articles. The annual publication trend exhibited a period of stability (prior to 2020) followed by rapid growth (from 2021 onwards), indicating the emerging prominence of virus and neuroinflammation research as a burgeoning field. The surge in research activity can be attributed to the heightened recognition of viral involvement in CNS-related disorders, which has been further accentuated by the COVID-19 outbreak-induced alterations in neural biochemical functions.

The bibliometric analysis revealed that the relevant publications were mainly published by corresponding authors from the United States, China, Germany, the United Kingdom, Canada, and Italy. This national disparity can be attributed to the strong correlation between academic capacity and a country’s economic situation. With robust government economic support, science and technology will continue to innovate and advance. Since the Renaissance, European scientists have embarked on exploring the fundamental structure and function of the nervous system, its interplay with psychology, as well as treatments for neurological disorders. In recent times, researchers in Germany, France, the United Kingdom, and the United States have employed cutting-edge imaging techniques such as electron microscopy to unravel the intricacies of nervous system architecture and functionality. In contemporary society, luminaries like Georg Nagel, Karl Deisseroth, Edward Boyden, Armbruster Roth et al., have pioneered optogenetics and chemogenetics respectively to precisely modulate neuronal activity. By integrating these approaches with brain-computer interfaces (BCIs), a deeper comprehension of distinct neuron roles in behavior modulation, learning processes,and memory formation has been achieved. Government investment in healthcare alongside scientific advancements serve as pivotal indicators of medical research output; factors that may contribute to why the United States leads in terms of publication quantity and citation impact. Whether analyzing the collaboration of countries, institutions, or authors on viral and neuroinflammation research also gravitates towards the United States—a testament to its remarkable contributions within this academic domain.This observation further underscores that other nations,institutions,and scholars should foster international cooperation to augment their influence.

Among the top 10 authors, three authors, Power C, Lane TE, and Buch s, have made the most outstanding contributions in this field. Professor Berman JW from the Albert Einstein College of Medicine is the author with the highest average number of citations per article (N= 55.17). His research focuses on the increased sensitivity of HIV infection of CD14^+^CD16^+^ monocytes to CCL2 and the expression of CCR2, JAM-A, and ALCAM on CD14^+^CD16^+^ monocytes promote the entry of HIV-infected and uninfected CD14^+^CD16^+^ monocytes into the brain (35). This in turn leads to ongoing neuroinflammation that occurs during HIV pathogenesis. Meanwhile, his findings indicate that CCR2 on CD14^+^CD16^+^ monocytes is a novel peripheral blood biomarker of HAND (30). Despite the significant contributions made by numerous authors in this field, it remains imperative to enhance collaboration among international and domestic institutions and laboratories in order to expedite the resolution of major scientific challenges and collectively achieve groundbreaking advancements from scratch. The majority of the top 10 journals that exhibit high research activity in viruses and neuroinflammation are published in the United States and Europe. The Journal of Neuroinflammation emerged as the journal with the highest number of publications, whereas Plos One garnered the highest number of average citations. In contrast, although China and India are also major contributors to research in this field, there is a lack of Asian publishers. This highlights the significance for Asia to establish its own foundations and develop internationally influential journals. **Figure 5C** demonstrates the widespread recognition and study of viruses in both basic and clinical research on neuroinflammation, which holds great importance for diagnosing and treating CNS-related diseases.

### 4.2 Hotspots and frontiers

The analysis of highly cited articles and frequent keywords provides valuable insights into influential findings, which can effectively guide future research directions in a specific field. In this study, we employed key co-occurrence analysis to identify the primary research focuses and emerging trends in virus-related neuroinflammation, as well as to elucidate the evolution and dynamics of its thematic structure.

Analysis of the references showed that 8 of the top 10 most cited articles were clinical trial articles and two reviews. These publications primarily focused on HAND, HIV-associated dementia (HAD), and COVID-19, reflecting the relatively concentrated research directions of virus and neuroinflammation, and their close clinical relevance provides scientific significance for basic research.

Keywords analysis of research hotspots showed that research on virus and neuroinflammation were mostly related to neurodegenerative diseases. Neurodegenerative diseases are characterized by the degeneration of various brain regions; however, they all exhibit two common features: neuroinflammation and an epidemiological association with viral infection. MS is a chronic inflammatory demyelinating disease of the central nervous system, in which the immune system plays a crucial role in its pathogenesis (36). Nerve fibers are enveloped by myelin sheaths, and the immune system erroneously targets these sheaths, resulting in nerve function impairment and subsequent disruption of normal nerve signal transmission, leading to associated symptoms (37). Viral infection is recognized as one of the primary triggers for MS development. Researchers have observed a 32-fold increase in MS risk following Epstein-Barr virus (EBV) infection (38). It was found that high levels of EBNA_386-405_ specific antibodies and GlialCAM_370-389_ derived from the CNS cross-react due to their similar structure, thus provoking the immune system to attack the body’s own nervous system and causing MS (39). Researchers have identified certain genetic factors and specific EBV variants that influence MS development by significantly attenuating immune responses against autoreactivity, thus promoting disease progression (39). Furthermore, AD and PD are also research hotspots in this field. In a study by Cairns DM, it was found that Varicella zoster virus (VZV) infection of cells quiescently infected with herpes simplex virus type 1 (HSV-1) causes reactivation of HSV-1 and consequent AD-like changes, including Aβ and P-tau accumulation (40). Moreover, the N protein of SARS-CoV-2 can interact with α-synuclein to accelerate the formation of amyloid deposits (41). Additionally, COVID-19-induced systemic inflammatory response or toxicity may exacerbate symptoms in elderly or severe PD patients; however, current clinical evidence does not establish a causal relationship between inflammation and PD (42). Since its first reported case in 2019, research on SARS-CoV-2 has remained active. The inflammatory response triggered by the virus can indirectly harm the nervous system, potentially leading to encephalitis, acute myelitis, cerebrovascular diseases, demyelinating diseases of the central nervous system, epilepsy, and other neurological disorders (43, 44). In conclusion, viral infection-induced neuroinflammation is involved in an increasing number of neurological diseases, thereby establishing a solid foundation for subsequent investigations. However, there remains a paucity of clinical studies targeting viral genes or proteins as therapeutic interventions for related neurological diseases. Consequently, the future focus in this field lies in the implementation of precise and efficacious personalized treatments that specifically target viruses to address neuroinflammation-related disorders.

### 4.3 Summary and prospects

**Figure 9:**
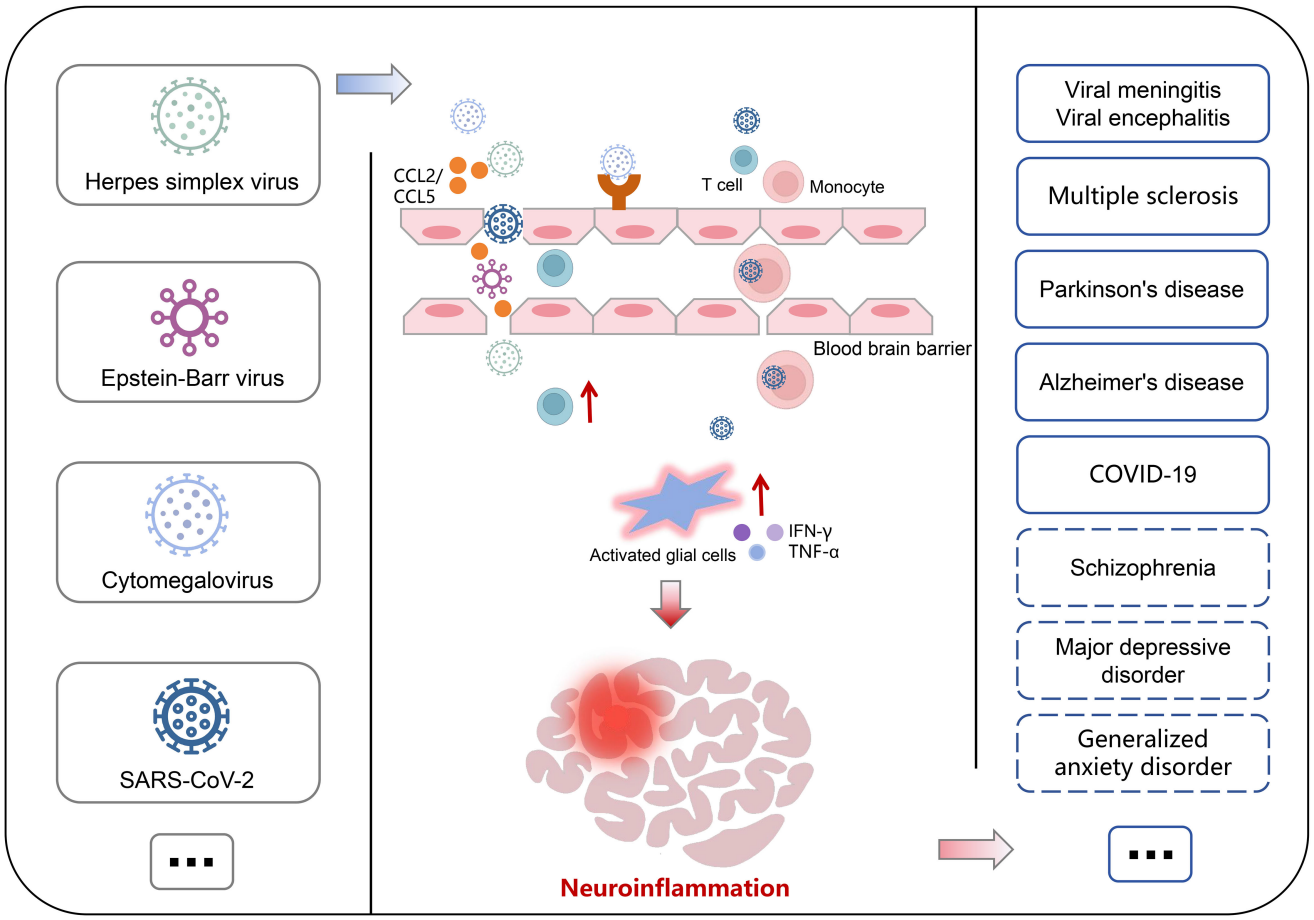
Association of virus and neurological disorders. The virus can directly traverse the endothelial cells of blood vessels and exploit the “Trojan horse” mechanism to breach the blood-brain barrier, thereby inducing an upregulation of immune cells and pro-inflammatory factors, facilitating excessive activation of glial cells for mediating neuroinflammation, consequently leading to neurological disorders. The contribution of this process in the pathogenesis of mental illnesses remains to be elucidated.

In summary, the involvement of immune cells and disruption of the BBB are crucial steps in the virus-induced neuroinflammatory process. Virus infection typically results in an upregulation of chemokines (CCL2 and CCL5) and alterations in tight junction protein expression, thereby modifying endothelial cell function and increasing vascular permeability. This allows for direct viral penetration across the initial barrier of the CNS. Simultaneously, viruses can also infect white blood cells (monocytes and macrophages), enabling them to transport pathogens across the BBB, leading to sustained infection of macrophages and microglia within the central nervous system. Prolonged viral infection induces a shift in microglial phenotype from a neuroprotective state to a neurodegenerative state, characterized by production of neurotoxins and activation of immune cells. Furthermore, upon invasion into the CNS, viruses induce accumulation of immune cells such as CD8^+^ cells as well as pro-inflammatory cytokines like IFN-γ and TNF-α. These factors can exacerbate inflammation within the CNS, ultimately contributing to onset of neurological diseases (**Figure 9**).

With the advancement of research on viral infection-induced neuroinflammation in nervous system diseases, novel research directions in this field have gained prominence. In recent years, the investigation of neuroinflammation caused by viral infection in psychiatric disorders has emerged as a primary area of study, garnering significant attention from researchers. Particularly since the onset of COVID-19, an emerging acute infectious disease capable of inducing not only systemic symptoms but also psychiatric disorders (45). The largest study to date on the health of COVID-19 patients, which encompassed data from over 236,000 individuals afflicted with COVID-19, has been published online in The Lancet Psychiatry (46). Findings revealed that within six months of contracting COVID-19, 33.62% of patients received a diagnosis for a neurological or psychiatric disorder, with 12.84% being newly diagnosed cases. Among critically ill patients, 46.42% were diagnosed with neurological or mental disorders, and among them, 25.79% were newly diagnosed cases. Anxiety (17.39%) and mental disorders (1.40%) emerged as the most prevalent mental health conditions observed in this cohort. Notably, individuals affected by COVID-19 exhibited a 44% higher risk of developing neurological and psychiatric conditions compared to those infected with influenza and a 16% higher risk compared to those suffering from respiratory infections (46). These results indicate that COVID-19 confers an elevated susceptibility to neurological and psychiatric disorders when contrasted against influenza and respiratory infections alike. Furthermore, depression represents a significant public health concern; recent reports suggest its association with disease progression as well as increased complications arising from specific viral infections such as SARS-CoV-2 and HIV infection while also rendering individuals more susceptible to viral infections (47, 48). revealed a decrease in the expression of Abelson helper integration site 1 (AHI1) in peripheral blood mononuclear cells (PBMC) and macrophages from individuals with major depression (MDD), resulting in impaired antiviral immune response (49). The hypothesis of inflammation in the development of schizophrenia suggests that maternal exposure to infections such as influenza virus, Toxoplasma, herpes simplex virus, measles virus, rubella virus during the first and second trimesters of pregnancy is considered an important risk factor for adult-onset schizophrenia (50). Furthermore, dormant human cytomegalovirus (CMV) infection may be associated with mood disorders, suicidal behavior, and neuroinflammation (51, 52). A higher proportion of individuals with psychiatric disorders were found to be CMV seropositive compared to controls. Interestingly, CMV seropositivity was also linked to increased microglial activity, suggesting that CMV-associated neuroinflammation could potentially contribute to psychiatric disorders (51). These studies collectively suggest that neuroinflammation caused by viral infection not only plays a role in neuroinflammatory diseases, but also may have a potential role in non-inflammatory neurological diseases. In this bibliometric analysis, although the number of such studies is limited, they have been increasing steadily over the years. The majority of publications focus on relevance studies; therefore, future research should aim to investigate specific mechanisms in greater detail. Moreover, considering that psychiatric disorder is a multifactorial and highly heterogeneous disease, it would be worthwhile to explore whether personalized antiviral treatment could serve as a potential therapeutic approach.

## 5 Limitations

This study represents the pioneering attempt to investigate the evolving trends and potential research frontiers on virus-induced neuroinflammation, employing a bibliometric approach. Nevertheless, certain limitations should be acknowledged in this investigation. Specifically, only English articles and reviews from the WOSCC database were included with a literature collection cut-off date of January 31, 2024, potentially excluding relevant publications. Moreover, it is worth noting that an article’s impact can be influenced by its publication duration; thus, some recently published high-quality articles might have been overlooked due to their low citation frequency. However, these limitations are unlikely to alter the fundamental trends elucidated in this paper.

## 6 Conclusion

Through comprehensive bibliometric analysis in virus and neuroinflammation, this study evaluates the publications information of different years, countries, institutions, authors, and journals and analyzes the theme development and future research hotspots. The findings indicate a growing research interest in the field. European and American countries have played a pivotal role in conducting extensive studies on this topic; however, China has also made significant contributions despite its limited impact thus far. Our findings reveal that multiple sclerosis, Parkinson’s disease, blood-brain barrier, COVID-19, Alzheimer’s disease, neurocognitive disorders, gene therapy, depression, maternal immune activation, gut microbiota, and stress are the primary focus and hot spots of this field. However, the majority of previous studies have primarily focused on investigating the association between viruses and neuroinflammatory diseases. Only a limited number of epidemiological studies have suggested that viral infections may also contribute to non-inflammatory neurological disorders, such as psychiatric conditions. By examining current research trends and emerging areas of interest in the field of viruses and neuroinflammation, this study aims to provide valuable insights into understanding the mechanisms underlying neuroinflammation induced by different types of viruses in relation to the onset and progression of psychiatric disorders.

## Data availability statement

The original contributions presented in the study are included in the article/**Supplementary Material**. Further inquiries can be directed to the corresponding author.

## Author contributions

DL: Data curation, Formal analysis, Investigation, Methodology, Software, Visualization, Writing – original draft, Writing – review & editing. MW: Conceptualization, Funding acquisition, Resources, Supervision, Writing – review & editing.
