# Supplementary material for: Potential value and research frontiers of virus in neuroinflammation: a bibliometric and visualized analysis
Source: Front Immunol. 2024 Jul 3;15:1390149. doi: 10.3389/fimmu.2024.1390149 (PMC11251911; doi:10.3389/fimmu.2024.1390149)
Supplement: Supplementary file 1 [file DataSheet_1.pdf]

Supplementary Material

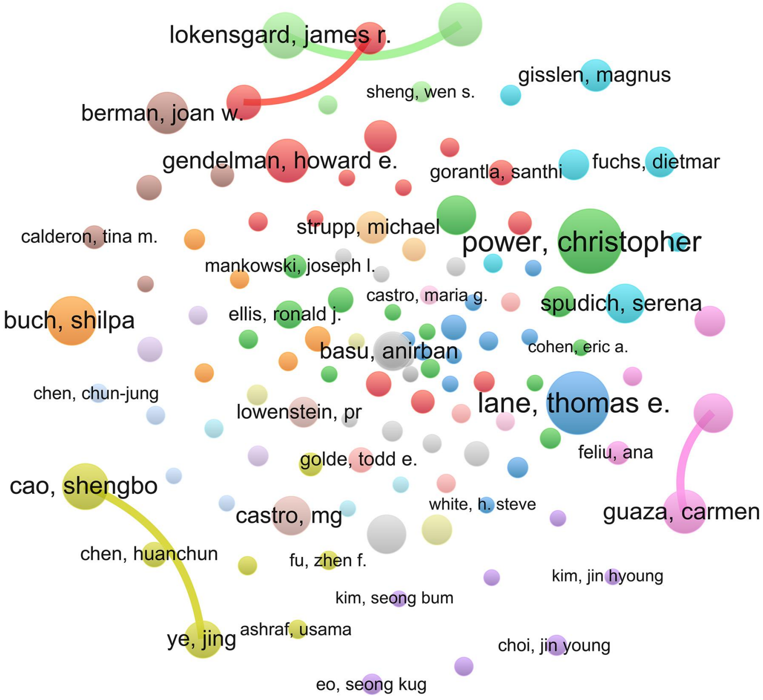

Supplementary Figure 1. Visual cluster analysis of cooperation among authors. The nodes of different colors represent the authors with different clusters, and the thickness of the lines indicates how closely authors cooperate.
